# Common Bean Baked Snack Consumption Reduces Apolipoprotein B-100 Levels: A Randomized Crossover Trial

**DOI:** 10.3390/nu13113898

**Published:** 2021-10-29

**Authors:** Alejandro Escobedo, Edgar A. Rivera-León, Claudia Luévano-Contreras, Judith E. Urías-Silvas, Diego A. Luna-Vital, Norma Morales-Hernández, Luis Mojica

**Affiliations:** 1 Tecnología Alimentaria, Centro de Investigación y Asistencia en Tecnología y Diseño del Estado de Jalisco (CIATEJ), A.C., Camino Arenero #1227 Col. El Bajío, Zapopan 45019, Mexico; aescobedoa@ciatej.mx (A.E.); jurias@ciatej.mx (J.E.U.-S.); nmorales@ciatej.mx (N.M.-H.); 2 Department of Molecular Biology and Genomics, University of Guadalajara (UdeG), Guadalajara 44350, Mexico; edgar.rleon@academicos.udg.mx; 3 Department of Medical Sciences, University of Guanajuato, Leon 37320, Mexico; c.luevanocontreras@ugto.mx; 4 Tecnologico de Monterrey, School of Engineering and Science, Av. Eugenio Garza Sada 2501 Sur, Monterrey 64849, Mexico; dieluna@tec.mx

**Keywords:** functional food, clinical trial, pulses, legume, sensory evaluation, apoB-100, cholesterol, triglycerides, phytosterol, saponin

## Abstract

Snack alternatives based on common beans (*Phaseolus vulgaris* L.) have been developed to promote pulse consumption. The purpose of this study was to evaluate the chemical composition, sensory acceptance and the effect of common bean baked snack (CBBS) consumption on blood lipid levels in participants with overweight and altered blood lipid levels. A sensory evaluation by 80 untrained judges was carried out using a hedonic scale. A randomized crossover 2 × 2 trial was performed, where 20 participants with overweight and one blood lipid alteration consumed 32 g of CBBS or did not consume it (control) for four weeks. Blood samples were taken to quantify the triglycerides, total cholesterol, LDL-c, HDL-c, ApoB-100, glucose and insulin. Furthermore, anthropometric, dietary and physical activity parameters were recorded. The overall acceptance of CBBS was similar compared to popcorn (*p* > 0.05). The consumption of CBBS reduced the apolipoprotein B-100 levels (*p* = 0.008). This reduction could be associated with the additional dietary fiber consumption during the CBBS period (*p* = 0.04). Although it did not improve any other blood lipid or glucose parameters (*p* > 0.05), it did not affect them either, which means that the CBBS could be consumed without compromising cardiovascular health.

## 1. Introduction

It is well-established that reduction in cardiovascular disease risk is proportional to the absolute change in low-density lipoprotein-cholesterol (LDL-c) and other apolipoprotein B-containing lipoproteins levels [1]. The energy imbalance between a high-energy diet and low physical activity, along with high consumption of saturated and trans fats, refined sugars and low dietary fiber consumption, increases cardiovascular disease development [2]. Regular exercise and healthy eating patterns, including fruit, vegetables and pulses in the daily diet, can reduce blood lipid concentrations.

Common bean (*Phaseolus vulgaris* L.) consumption is associated with multiple health benefits, including preventing and managing obesity, type 2 diabetes, cancer and cardiovascular diseases [3,4,5]. Beans represent an inexpensive source of bioactive compounds such as proteins, dietary fiber, linoleic and oleic acids, polyphenols, saponins and phytosterols [6]. Despite these benefits and the extensive population growth, common bean intake per capita has remained stagnant in the last 30 years [7]. 

In recent years, snack alternatives based on common beans have been developed to promote pulse consumption [8]. Meanwhile, consumers are changing their food preferences, choosing certain food products to obtain health benefits, and pulse-based snacks could satisfy this growing demand [9]. However, these snacks must resolve particular challenges. Common bean-based snacks must present good acceptability from regular snack consumers to ensure commercial success. Furthermore, the concentration of α-galactooligosaccharides must be reduced to avoid unpleasant effects, including flatulence, particularly in people with gastrointestinal sensibility [10]. Finally, snack evaluation cannot be limited to assaying the nutritional composition and consumer acceptance. The snacks’ health benefits must be scientifically validated to proclaim them as functional foods. Randomized clinical trials are considered the mandatory gold standard to demonstrate their functionality [11,12]. 

The purpose of this study was to evaluate the chemical composition, sensory acceptance and the effect of common bean baked snack (CBBS) consumption on blood lipid levels in people with overweight and altered blood lipid levels. It was hypothesized that consuming 32 g of the common bean baked snack over four weeks reduces blood lipid levels in people with overweight and altered blood lipid levels.

## 2. Materials and Methods

### 2.1. Snack Preparation

**Figure 1:**
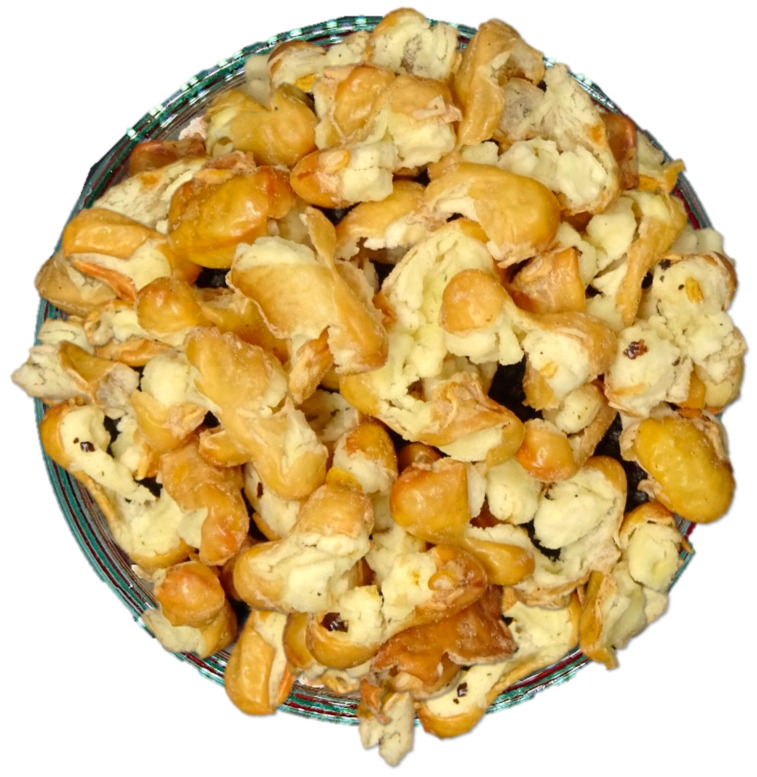
Common bean baked snack.

Peruano beans (*Phaseolus vulgaris* L.), salt and citric acid were purchased at a local market, and the beans were cleaned to remove debris and damaged seeds. The common beans were soaked in purified water. The soaking water was removed, and the beans were rinsed and cooked. Salt and citric acid were added to the cooked beans before baking (patent pending) (Figure 1) [13]. Then, the common bean baked snacks were individually packaged in resealable bags and stored at room temperature.

### 2.2. Chemical Composition

The snack was analyzed for total protein (AOAC method 979.09), soluble and insoluble dietary fiber (AOAC method 991.43), total fat (AOAC method 2003.06), saturated fat (AOAC method 996.06), sugars (AOAC method 920.183), available carbohydrates (calculated by difference) and sodium (INS-SM-US-71) content. Resistant starch (K-RSTAR 08/18) and α-galactooligosaccharides (K-RAFGL 04/18) were quantified following the specifications of the assay kits from Megazyme (Wicklow, Ireland). 

Total polyphenols were analyzed using an adaptation of the Folin–Ciocalteu method described by Hsieh-Lo et al. [14]. Methanol was utilized for polyphenol extraction as it is a commonly used organic solvent with affine polarity for most phenolic compounds, which improves the extraction efficiency [15]. Methanolic extracts were prepared by mixing 100 mg of the ground sample (0.59 mm sieve) with 900 µL of 99% methanol. The solutions were mixed and sonicated at 20 °C and 37 kHz for 30 min. The samples were centrifuged at 3000× *g* for 10 min. The supernatants were decanted into new microfuge tubes and dried using a centrifugal vacuum concentrator at 1000× *g* for 30 min. The samples were reconstituted with 1 mL of distilled water, then 50 μL were placed in a 96-well flat-bottom plate with 50 μL of the Folin–Ciocalteu reagent and allowed to stand for 5 min. Finally, 100 µL of a 20% sodium carbonate solution (*w*/*v*) were added and incubated for ten more minutes. The absorbance was read at 765 nm, and the results were expressed as gallic acid equivalents (GAE).

Saponin extraction was performed based on the method proposed by Guo et al. [16]. The samples were milled and sieved with a 0.59 mm sieve. Then, 50 mg of sample powders were weighed accurately into a microcentrifuge tube, and 1 mL of 70% ethanol was added. The tubes were mixed and sonicated at 20 °C and 37 kHz for 30 min. Finally, the tubes were centrifugated at 3000× *g* for 10 min. Meanwhile, saponin content was determined using the method of Lai et al. [17] with slight modifications. Briefly, 50 μL of the extract were mixed with 50 μL of freshly prepared 8% vanillin solution (in ethanol) and 500 μL of 72% sulfuric acid in an ice water bath. The mixture was warmed in a water bath at 60 °C for 10 min and then cooled in ice-cold water. The absorbance was read at 544 nm, and the results were expressed as milligrams of soyasaponin Bb equivalents (SBE). Soyasaponin Bb reference substance was acquired from Phyproof (PHL83545, PhytoLab, Germany).

Phytosterol extraction was carried out employing a method used by Saptarini et al. [18]. First, sample powders in the amount of 1 g were weighed accurately into 15 mL centrifuge tubes, and 3 mL of *n*-hexane and 70% ethanol (82:18) solution were added. The tubes were mixed and sonicated at 20 °C and 37 kHz for 30 min. The tubes were centrifugated at 3000× *g* for 10 min, and the supernatant was recovered in a 22 mL glass vial. Next, 1 mL of 26.73 M KOH was added to the extract, mixed, boiled (100 °C) for 1 h and cooled at room temperature. The mixture was transferred to a 50 mL separatory funnel, and the vial was washed with a 2 mL portion of hot water and another of room temperature water. The mixture was extracted four times with 5 mL portions of *n*-hexane. Each portion was collected into a test tube, and *n*-hexane was evaporated on a rotary evaporator at 40 °C and 260 mBar. Then, the concentration of phytosterols was measured using a method proposed by Saptarini et al. [19]. The dried phytosterol extract was dissolved with 1 mL of chloroform. Later, 150 μL of the extract and standard solutions were added into 1.5 mL microcentrifuge tubes, and they were mixed with 300 μL of the Liebermann–Burchard reagent and 300 μL of chloroform. The solutions were covered with aluminum and incubated in the dark for 10 min, and absorbance was read at 630 nm. The Liebermann–Burchard reagent was prepared by cooling acetic anhydride for 30 min in an ice bucket, and 10 mL were mixed with 1 mL of concentrated sulfuric acid. The results were expressed as milligrams of β-sitosterol equivalents (β-SE). The β-sitosterol (S1270) reference substance was acquired from Sigma-Aldrich (S1270, St. Louis, MO, USA).

### 2.3. Texture and Sensory Evaluation

The crispness and hardness properties of the common bean baked snack (CBBS) were compared with commercial snacks, including pork rinds (Sabritas^®^), banana chips (BEL ARA^®^), popcorn (naturally flavored ACT II^®^), corn extrudates (Cheetos^®^ crunchy cheese-flavored snacks), fried corn (BEL ARA^®^) and fried peas (BEL ARA^®^). Crispness was determined with a five-bladed Kramer shear cell, hardness—with a 100 mm compression platen using a TA.TX.Plus Texture Analyser (Stable Micro Systems, UK). Six replicates were performed for each snack for the crispness analysis and 25 for the hardness analysis. 

Subsequently, the CBBS was evaluated for overall acceptance, appearance, crispness and hardness by 80 untrained consumers using a five-point hedonic scale as follows: 5—like very much, 4—like, 3—neither like nor dislike, 2—dislike, 1—dislike very much. Additionally, corn extrudates (Cheetos^®^ crunchy cheese-flavored snacks) and popcorn (naturally flavored ACT II^®^) were also assessed as they presented similar hardness and crispness values to the CBBS (*p* > 0.05). The volunteers were recruited from the Center for Research and Assistance in Technology and Design of the State of Jalisco and social media. The laboratory temperature was kept between 20 and 22 °C, the lights were on, and the doors were closed.

### 2.4. Clinical Trial

#### 2.4.1. Trial Design and Participants

The study consisted of a randomized crossover study of four-week intervention and control periods with a four-week washout period. Eligible participants were adult men and women aged 18–40 years with a body mass index (BMI) between 25.0 and 29.9 kg/m^2^ and the presence of one or more alterations of serum lipid levels, including total cholesterol (TC) ≥ 200 mg/dL, low-density lipoprotein-cholesterol (LDL-c) in the range from 100 to 190 mg/dL, high-density lipoprotein-cholesterol (HDL-c) < 50 mg/dL in females and <40 mg/dL in males and triglycerides (TG) in the range from 150 to 500 mg/dL. Participants were not included if they had one of the following conditions: pregnancy, lactation, established plans to lose or gain weight in the next three months, modification of diet or physical activity in the last three months, diagnosis of diabetes, cancer, cardiovascular disease, gastrointestinal disorder, pancreatitis, kidney, liver or thyroid disease, smoking or drug use, sensitivity for common bean consumption and pharmacological treatment or consumption of nonprescription drugs, herbal or nutritional supplements known to modify serum lipid levels. The participants were recruited by open invitation in Guadalajara, Mexico. The study was carried out at two centers, the University Center for Health Science of the University of Guadalajara and the Center for Research and Assistance in Technology and Design of the State of Jalisco, from May 2021 to September 2021. This trial was registered at clinicaltrials.gov under No. NCT05028699.

#### 2.4.2. Interventions

The interested individuals were screened based on a self-administered online form and lipid blood profile results to establish eligibility. 

**Figure 2:**
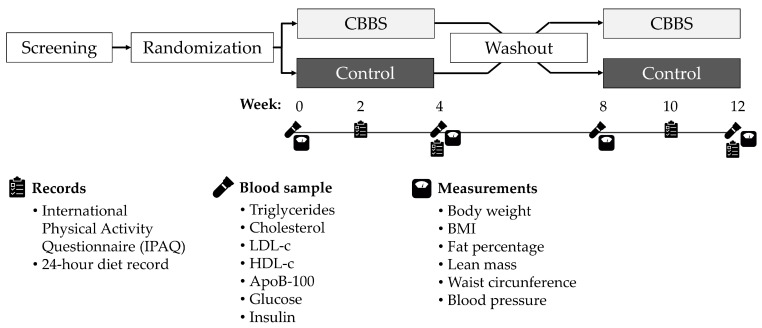
Study design diagram.

The recruited subjects were assigned using a simple randomization method to the intervention group or the control group. The randomization sequences for the participants were computer-generated. The participants were asked to consume 32 g of the common bean baked snack daily for four weeks during the intervention period (Figure 2). Meanwhile, they received no intervention in the control period. We decided not to use a placebo because no placebo for this common bean baked snack could achieve successful blinding of the participants. The participants were instructed to maintain their usual physical activity levels and diet behaviors during the intervention, control and washout periods. 

#### 2.4.3. Primary and Secondary Outcomes

The primary outcome was a change of the biomarkers directly related to lipid metabolism, including TG, TC, LDL-c, HDL-c, ApoB-100 and non-HDL cholesterol, after the CBBS consumption period. Meanwhile, the secondary outcomes were changes in blood glucose and insulin levels, blood pressure, body weight, body fat percentage, muscle mass, waist and hip circumferences and dietary and physical activity habits.

#### 2.4.4. Outcome Measurements

Blood samples were collected after eight-hour fasting at the beginning and the end of each period. After separation by centrifugation, serum was aliquoted and stored at −80 °C until the end of the study. The TC, TG, HDL-c and glucose levels were quantified on a Vitros 350 Analyzer (Ortho Clinical Diagnostics, Inc., Raritan, NJ, USA) using multilayered slides, analytical elements coated on a polyester support. These analyses were based on enzymatic methods described by Spayd et al. [20], Allain et al. [21] and Trinder [22]. The LDL-c was determined using the Friedewald formula [23]. Apolipoprotein B-100 was determined employing an ELISA kit (RAB0610, Sigma-Aldrich, St. Louis, MO, USA). Insulin was analyzed using an ARCHITECT i2000SR immunoassay analyzer (Abbott, Chicago, IL, USA), and according to the Matthews formula, the homeostatic model assessment of insulin resistance (HOMA-IR) was calculated [24].

Body weight, body fat percentage and muscle mass were obtained using a bioelectrical impedance scale (BC-585F, Tanita Corporation, Tokyo, Japan), and height was measured using a Seca 213 stadiometer (Medical Measuring Systems and Scales Seca, Hamburg, Germany). Waist and hip circumferences were measured according to the International Society for the Advancement of Kinanthropometry standards. Blood pressure was recorded using an ambulatory blood pressure monitor (HEM-7600, Omron Healthcare, Inc., Kyoto, Japan). 

The participants completed 24-h diet records of one weekday in the second week and the fourth week of each period. The physical activity level was assessed using the Short Form of the International Physical Activity Questionnaire with the same frequency.

### 2.5. Statistical Analysis

A total of 20 participants were estimated to detect a 20% change in LDL-c, assuming a within-subject SD of 27%, at a 5% significance level with 80% power. Furthermore, four more subjects were added to the sample size to account for dropouts.

Statistical analyses were performed using NCSS Statistical Analysis and Graphics v21.0.3 (NCSS, LLC., Kaysville, UT, USA). All the variables were assessed for normality, and the values were presented as the means ± SD or the medians (IQRs). Categorical variables such as sensory outcomes were analyzed using the Kruskal–Wallis test to identify significant differences between scores. Quantitative variables, including clinical, biochemical, dietary and physical activity data, were compared using the paired sample *t*-test or the Wilcoxon rank-sum test according to their distribution. In addition, carryover effects were evaluated using the paired sample *t*-test or the Wilcoxon rank-sum test by comparing the baseline data from both groups and sequences.

## 3. Results and Discussion

### 3.1. Chemical Composition

The chemical composition of the common bean baked snack (CBBS) is illustrated in Table 1. The snack contains high levels of protein, similarly to other extruded common bean-based snacks. For example, Ai et al. [25] observed that the protein concentration ranged from 19.6 to 27.7% in four common bean varieties extruded under eight different conditions. In contrast, the protein content was higher compared to deep-fat fried pulse-based snacks. Vasundhra et al. [26] prepared three deep fat-fried extrudates with a mixture of field beans or red or white cowpeas with cornstarch and mashed potatoes, and they found protein levels to be between 9.49 and 17.18%.

Dietary fiber is one of the major components that help to control lipid blood levels. The CBBS contains 16.1% of total dietary fiber, of which 3.5% is soluble fiber, and 12.6% is insoluble fiber. The high dietary fiber content is a common characteristic of pulse-based snacks. For instance, Flores-Silva et al. [27] formulated an extruded and deep fat-fried snack composed of chickpea flour (60%), maize flour (30%) and unripe plantain flour (10%), and it contained 18.2% of total dietary fiber and 3.3% of resistant starch. Conversely, the CBBS dietary fiber content is superior to several commercial snacks. For example, Cheetos^®^ and ACT II^®^ naturally flavored popcorn snacks claim to contain 1.3% and 10.0% dietary fiber, respectively. Furthermore, their fat content ranges between 26.0 (popcorn) and 36.7% (Cheetos^®^), higher than the 1.6% of the CBBS. Naturally, snacks similar to the CBBS that are deep fat-fried instead of baked contain elevated fat concentrations. Bozdemir et al. [28] developed three different whole chickpea deep fat-fried snacks with fat contents ranging from 13.5 to 13.9%. 

Although α-galactooligosaccharides (α-GOS) can confer some benefits for gastrointestinal health, they could also produce unpleasant effects such as flatulence, therefore decreasing acceptance. The combination of soaking, cooking and baking procedures reduced the α-GOS content of the CBBS by 60.7% (data not shown). Based on previous studies, the phytochemical concentration, including polyphenols, saponins and phytosterols, decreased as expected [29,30,31].

### 3.2. Sensory Evaluation

**Figure 3:**
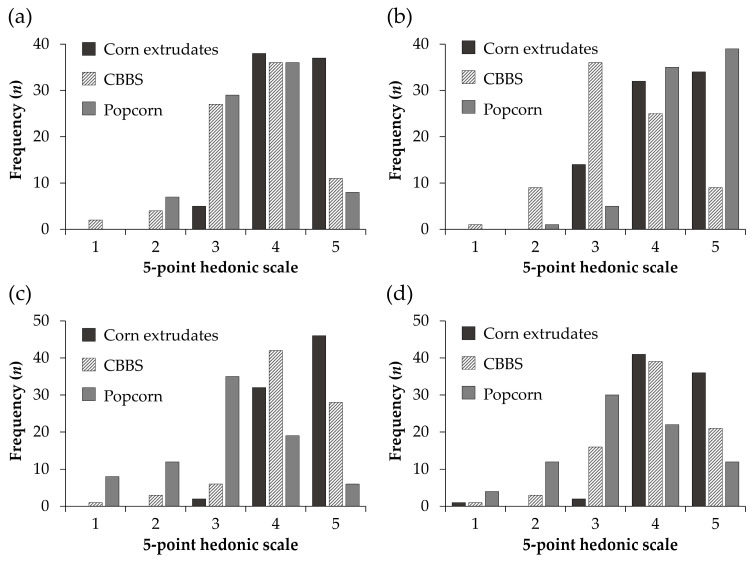
Frequency distribution of sensory evaluation outcomes of the common bean baked snack (CBBS), ACT II^®^ naturally flavored popcorn and crunchy Cheetos^®^. (**a**) Overall acceptance; (**b**) appearance; (**c**) crispness; (**d**) hardness.

The crispness and hardness outcomes are presented in Figure S1. The results demonstrated that the CBBS had acceptable sensory attributes. The overall acceptance of the CBBS was similar compared to popcorn (*p* > 0.05), with a median of four, indicating that consumers liked both snacks (Figure 3a). In contrast, corn extrudates were the most accepted snack (*p* < 0.05), probably because they were the only ones flavored. Despite the lack of flavoring, the CBBS showed high acceptability. Common bean-based snacks have acceptance slightly above the midpoint of hedonic scales. For instance, Ramírez-Jiménez et al. [32] elaborated a snack bar with cooked bean flour and oat flour and scored five using a nine-point scale. Similarly, baked chips prepared with pinto bean and faba bean flours had a mean score of 6.46 on a nine-point scale [33]. 

The appearance was majorly rated three (“Neither like nor dislike”) for the CBBS (Figure 3b). The panelists suggested that the appearance can be improved by adding colorful flavorings. Regarding the texture, the liking of the crispness was higher for the CBBS than for popcorn (*p* < 0.05) but the same compared to corn extrudates (*p* > 0.05) (Figure 3c). These similarities between the CBBS and corn extrudates are consistent with the crispness results of the texture analysis, where they presented equivalent crispness values (*p* > 0.05) (Figure S1). According to the frequency distribution, 87.5% of the judges scored the crispness acceptability with four or five, meaning that the CBBS crispness was highly accepted. Meanwhile, consumers liked much the hardness of the CBBS, which was not different from the other snacks (*p* > 0.05) (Figure 3d).

### 3.3. Clinical Trial

**Figure 4:**
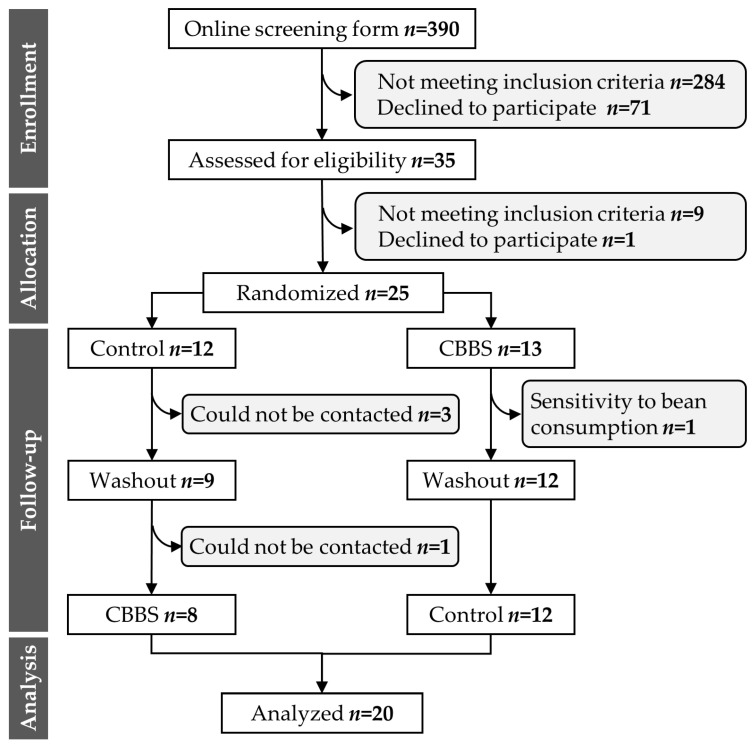
Participant flow diagram. CBBS, common bean baked snack.

A total of 390 candidates answered the online screening form. Twenty-five participants were randomly assigned, and twenty completed the study. Four were impossible to contact, and one subject dropped out due to sensitivity to the CBBS consumption (Figure 4). The baseline characteristics are shown in Table 2.

#### 3.3.1. Anthropometric, Dietary and Physical Activity Records

There were no significant changes and carryover effects among both groups in anthropometric and blood pressure measurements (Table S1). In chronic studies, linoleic acid supplementation could negatively affect body weight; however, the amount of linoleic acid in the CBBS dose was not enough to observe any body weight modification. Besides, the last eight weeks reported in studies in animal models compared to the four weeks in this study [34]. Energy and nutrient intake did not differ between the groups, but the total dietary fiber did (Table 3). The intake of polyphenols, saponins and phytosterols was omitted due to the lack of information in the food composition data. Meanwhile, physical activity seemed higher in the control group than in the CBBS group, but no significant difference was shown (*p* = 0.10). 

#### 3.3.2. Biochemical Analyses

The mean responses of biomarkers during the CBBS and control periods are shown in Table 4. There were no significant differences in most blood lipid parameters, including triglycerides (TG), total cholesterol (TC), HDL-c, non-HDL-c and LDL-c. Previous studies with pulse-based snacks obtained similar results. For instance, Marinangeli et al. [35] compared the consumption of three different muffins containing whole pea flour, pea hulls or wheat flour for their ability to reduce cardiovascular risk factors in a four-week crossover trial with twenty-three subjects with overweight and hypercholesterolemia. The daily doses of whole pea flour and pea hulls were approximately 50 g/day. They observed no reductions in the within-group lipid concentrations for TG, TC, LDL-c and HDL-c.

Similarly, Cryne et al. [36] found no differences in the same parameters after evaluating the effect of chickpea, lentil and pea flours and potato flakes. The study was a crossover trial with twenty-one healthy males who consumed 100 g of pulse flours for 28 days each. They had the freedom to incorporate them into hummus-like spreads, pancakes or shakes. In another study, Ramírez-Jiménez et al. [37] assessed the metabolic impact of the daily intake of 50 g of a common bean and oat snack bar on reducing hypertriglyceridemia markers in females. They conducted a parallel trial with 26 participants for eight weeks, and they noted that the TG levels were decreased, but the TC, HDL-c and LDL-c levels were not. As most of them stated, more considerable doses were probably needed to observe changes in these blood lipid parameters. However, the dose was higher than the U.S. Department of Agriculture’s suggested daily serving size of 1.5 cups of cooked pulses per week, but not high enough to be a reasonable portion size [38].

On the other hand, the ApoB-100 concentration was significantly reduced during the CBBS period. To our knowledge, no study assessing a pulse-based snack has shown an ApoB-100 reduction, and only two studies have addressed the evaluation of ApoB levels in cooked pulses. A total of 46 females with overweight had a diet rich in brown beans (86 g), chickpeas (82 g) and kernel-based barley products (58 g) or a control diet for four weeks, and a significant reduction of ApoB was found [39]. In contrast, no changes in the ApoB concentrations were observed in a parallel trial with 134 females that consumed 750 mL of cooked pulses weekly for 16 weeks [40].

It was expected that ApoB-100 reduction would be accompanied by a decrease in LDL-c or TC. LDL particles can be classified according to size and density into small dense low-density lipoprotein (sdLDL) and large buoyant LDL [41]. The sdLDL is potentially atherogenic as its small particle size enables it to penetrate easily into the arterial wall. It could predict the risk for incident coronary heart disease even in subjects considered to be of low cardiovascular risk based on their LDL-c level [42]. The reason for this is that sdLDL particles are smaller and contain less cholesterol. Therefore, lowered levels of sdLDL also represent a decreased number of atherogenic particles, which may not be reflected by the levels of LDL-c [41]. This suggests that potentially there was a reduction of sdLDL particles and an increment of large buoyant LDL. However, a direct measurement of sdLDL-cholesterol is needed to confirm this hypothesis.

Common beans contain several components with cardioprotective properties, including dietary fiber, polyphenols, saponins and phytosterols. There are three different mechanisms whereby soluble fiber could lower cholesterol. Soluble and insoluble dietary fibers interact with bile acids impeding their reabsorption, increasing bile salt excretion in feces [43]. Furthermore, soluble fiber increases the production of short-chain fatty acids through fermentation in the colon. These fatty acids may interfere with endogenous TC production [44]. Finally, soluble fiber delays gastric emptiness, slowing glucose uptake. Consequently, it lowers insulin levels, which could potentially reduce hepatic TC synthesis [45].

Polyphenols may exert a TG-lowering mechanism by inhibiting the pancreatic lipase and interfering with intestinal absorption [46]. Diversely, saponins and phytosterols may interact with dietary and bile salt cholesterol, reducing their absorption by forming insoluble complexes [47,48]. Through several clinical trials, different-source phytosterols have proven to reduce the sdLDL levels [49]. However, the exact mechanism remains to be elucidated. It can be speculated that a combination of all these mechanisms could be responsible for ApoB-100 reduction.

As expected, blood glucose, insulin and HOMA-IR levels did not differ within subjects. At least 12 weeks of continuous snack consumption would be needed to observe a significant modification. These results showed that insulin sensitivity was not modified during the 12 weeks of the study. Therefore, there was no interference in lipid metabolism.

Although it had a crossover design, this study was limited by the sample size, and the outcomes should be replicated with a larger population. The duration of the study was established according to the European Food Safety Authority (EFSA) and the Canada Health regulations, where they suggest minimum intervention periods of 3–4 weeks for a visible change in blood lipids [50,51]. The EFSA also considers that studies should ideally be performed over eight weeks to observe a sustained effect. Nevertheless, the acceptability of a snack over a prolonged period could impact the subjects’ compliance over time [52]. Besides, the subjects were chosen with either elevated cholesterol-related biomarkers or elevated triglycerides, which are different metabolic conditions. Additionally, the absence of a placebo could influence the outcomes; however, it was not possible to use a placebo fully capable of blinding the participants. Finally, including the sdLDL and apolipoprotein A1 measurements would provide a more comprehensive understanding of the results.

## 4. Conclusions

In conclusion, the daily consumption of 32 g of a highly nutritious and sensory accepted common bean baked snack (CBBS) reduces the blood levels of apolipoprotein B-100; this could positively influence cardiovascular health. The additional dietary fiber intake supports this reduction. Although it did not improve any other blood lipid parameters, it did not negatively affect them, suggesting that the CBBS could be consumed without compromising cardiovascular health. Similar and more extensive studies need to be conducted on other populations to ratify its effectiveness. These findings should encourage the development of pulse-based snacks as they are healthier snack alternatives.

## Figures and Tables

**Table 1:** Chemical composition of the common bean baked snack ^1^.

Compound	Portion Size	
	100 g	32 g
Moisture (g)	2.8 ± 0.5	0.9 ± 0.2
Energy (kcal)	316	101
Proteins (g)	26.9 ± 0.2	8.6 ± 0.1
Available carbohydrates (g)	48.4 ± 1.7	15.5 ± 0.5
Total dietary fiber (g)	16.1 ± 0.6	5.2 ± 0.2
Soluble fiber (g)	3.5 ± 0.6	1.1 ± 0.2
α-GOS (g)	1.34 ± 0.04	0.43 ± 0.01
Insoluble fiber (g)	12.6 ± 0.7	4.0 ± 0.2
Resistant starch (g)	2.13 ± 0.1	0.68 ± 0.03
Total fat (g)	1.6 ± 0.2	0.5 ± 0.1
Saturated fat (g)	0.3 ± 0.1	0.1 ± 0.03
Sodium (mg)	654.7 ± 12.2	209.5 ± 3.9
Total polyphenols (mg GAE)	112.9 ± 0.7	36.1 ± 0.2
Saponins (mg SBE)	472.0 ± 13.9	151.1 ± 4.4
Phytosterols (mg β-SE)	33.6 ± 3.5	10.7 ± 1.1

^1^ The values are the means ± SD; α-GOS, α-galactooligosaccharides; GAE, gallic acid equivalents; SBE, soyasaponin Bb equivalents; β-SE, β-sitosterol equivalents.

**Table 2:** The baseline characteristics of the study participants ^1^.

Variables	Total (*n* = 20)
Age (y)	26.0 ± 4.9
Female/male (*n*)	9/11
BMI (kg/m^2^)	27.2 ± 1.2
Triglycerides (mg/dL)	178.7 ± 73.7
Total cholesterol (mg/dL)	160.8 ± 22.9
HDL-c (mg/dL)	43.2 ± 9.8
LDL-c (mg/dL)	81.9 ± 24.7

^1^ The values are the means ± SD. BMI, body mass index; HDL-c, high-density lipoprotein-cholesterol; LDL-c, low-density lipoprotein-cholesterol.

**Table 3:** Dietary intake and physical activity in the CBBS group and the control group ^1^.

Variables	CBBS	Control	*p* ^2^	
			Carryover (Sequence) ^3^	Treatment Effect
Energy intake (kcal)	2084 ± 626	1900 ± 759	0.35	0.29
Carbohydrates (g)	228 ± 82	221 ± 95	0.29	0.52
Total sugars (g)	83 ± 55	87 ± 59	0.36	0.66
Total dietary fiber (g)	22.9 (18.8–30.9)	18.8 (12.4–26.5)	0.66	0.04 *
Proteins (g)	103 ± 37	88 ± 41	0.71	0.25
Total fat (g)	81 ± 35	71 ± 35	0.50	0.50
Saturated fat (g)	26 ± 14	22 ± 11	0.51	0.49
Monounsaturated fat (g)	26 ± 14	22 ± 13	0.37	0.65
Polyunsaturated fat (g)	17 ± 10	15 ± 9	0.12	0.66
Cholesterol (mg)	360 ± 235	345 ± 257	0.88	0.79
Sodium (mg)	2308 ± 957	2167 ± 979	0.58	0.68
Physical activity (MET-h/wk)	17.8 ± 25.3	26.3 ± 27.4	0.17	0.10

^1^ The values are the means ± SD or the medians (IQRs) for nonnormally distributed variables; ^2^ *p*-values represent the significance of treatment and carryover effects using the paired sample *t*-test or the Wilcoxon rank-sum test; ^3^ *p*-values for sequence interaction. * indicates values that are significantly different (*p* < 0.05).

**Table 4:** Biochemical parameters of 4-week follow-up data of the CBBS group and the control group ^1^.

Variables	CBBS		Control		*p* ^2^		
	Baseline	4-Week	Baseline	4-Week	Carryover (Baseline) ^3^	Carryover (Sequence) ^4^	Treatment Effect
Triglycerides (mg/dL)	143.4 ± 84.1	147.9 ± 101.5	139.6 ± 82.0	148.0 ± 74.3	0.75	0.36	0.87
Total cholesterol (mg/dL)	148.8 ± 31.5	153.3 ± 34.0	154.4 ± 27.6	154.2 ± 32.4	0.46	0.26	0.79
HDL-c (mg/dL)	41.1 ± 14.1	41.1 ± 11.6	41.2 ± 9.4	42.0 ± 14.4	0.95	0.48	0.51
Non-HDL-c (mg/dL)	107.7 ± 25.8	112.3 ± 34.2	113.2 ± 27.8	112.2 ± 26.7	0.31	0.13	0.90
LDL-c (mg/dL)	78.9 ± 24.7	82.7 ± 28.3	85.2 ± 23.7	82.6 ± 25.3	0.29	0.26	0.81
ApoB-100 (mg/dL)	77.2 ± 22.1	56.6 ± 12.7	68.1 ± 27.9	74.2 ± 26.4	0.17	0.07	0.0084
Glucose (mg/dL)	88.9 ± 11.0	91.1 ± 9.6	90.2 ± 9.4	90.5 ± 8.1	0.68	0.70	0.93
Insulin (mU/L)	10.9 ± 5.3	12.8 ± 6.7	11.7 ± 5.8	11.3 ± 5.7	0.38	0.34	0.18
HOMA-IR	2.4 ± 1.4	2.9 ± 1.6	2.6 ± 1.3	2.6 ± 1.3	0.50	0.33	0.25

^1^ The values are the means ± SD; ^2^ *p*-values represent the significance of treatment and carryover effects using the paired sample *t*-test; ^3^ *p*-values for the CBBS baseline vs. the control baseline interaction; ^4^ *p*-values for sequence interaction. HDL-c, high-density lipoprotein-cholesterol; LDL-c, low-density lipoprotein-cholesterol; ApoB-100, apolipoprotein B-100; HOMA-IR, homeostatic model assessment of insulin resistance.

## Data Availability

The data presented in this study are available on request from the corresponding author.

## Supplementary Materials

The following are available online at https://www.mdpi.com/article/10.3390/nu13113898/s1, Figure S1: Textural properties of snacks, Table S1: Anthropometric and blood pressure measurements characteristics of baseline and 4-week follow-up of CBBS and control groups.
