# Supplementary material for: Common Bean Baked Snack Consumption Reduces Apolipoprotein B-100 Levels: A Randomized Crossover Trial
Source: Nutrients. 2021 Oct 29;13(11):3898. doi: 10.3390/nu13113898 (PMC8624358; doi:10.3390/nu13113898)
Supplement: Supplementary file 1 [file nutrients-13-03898-s001.zip › nutrients-1431574-supplementary.pdf]

Table S1. Anthropometric and blood pressure measurements characteristics of baseline and 4-week follow-up of CBBS and control groups. [1]

Variables	CBBS		Control		*P* values [2]		
	Baseline	4-wk	Baseline	4-wk	Carry-over (baseline) [3]	Carry-over (sequence) [4]	Treatment effect
Body weight (kg)	77.3 ± 12.1	77.8 ± 11.7	77.8 ± 11.6	77.8 ± 11.7	0.12	0.78	0.58
BMI (kg/m2)	27.2 ± 1.4	27.4 ± 1.2	27.4 ± 1.2	27.4 ± 1.2	0.11	0.50	0.54
Fat percentage (%)	29.0 ± 6.2	29.5 ± 6.8	29.0 ± 6.0	29.6 ± 6.5	0.92	0.50	0.86
Lean mass (kg)	52.3 ± 10.2	52.1 ± 10.0	52.5 ± 10.1	52.2 ± 10.1	0.34	0.89	0.90
Waist circumference (cm)	92.3 ± 10.5	92.7 ± 10.4	92.7 ± 10.3	92.8 ± 10.5	0.53	0.53	0.89
Hip circumference (cm)	104.6 ± 5.3	104.8 ± 5.2	104.9 ± 4.0	105.5 ± 4.4	0.62	0.64	0.16
Systolic BP (mm Hg)	111.0 ± 8.8	113.3 ± 10.4	112.8 ± 9.8	112.4 ± 9.2	0.37	0.73	0.62
Diastolic BP (mm Hg)	73.2 ± 7.7	72.2 ± 8.2	73.6 ± 9.4	74.0 ± 8.1	0.80	0.92	0.16

[1] Values are means ± SD. [2] *P* values represent the significance of treatment and carryover effects using a paired samples *t*-test. [3] *P* values for CBBS baseline × Control baseline interaction. [4] *P* values for sequence interaction. BMI, body mass index.

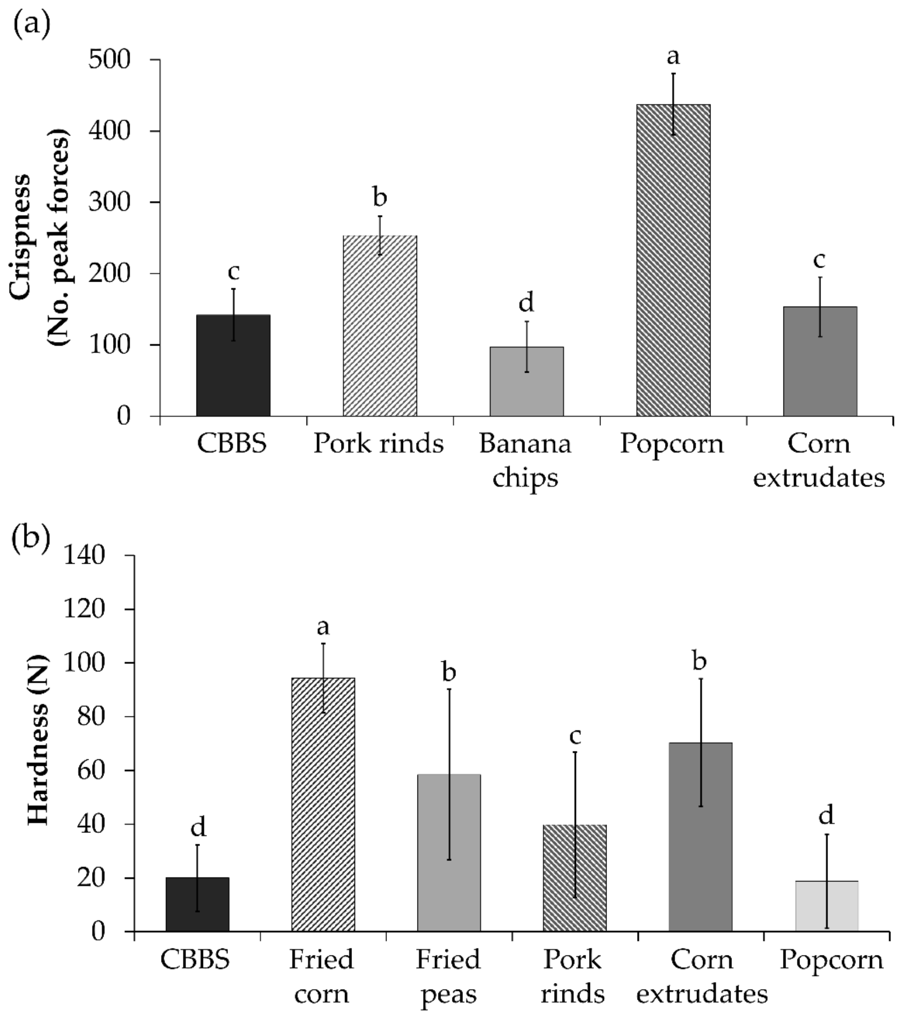

Figure S1. Textural properties of snacks. (**a**) Crispness; (**b**) Hardness. Mean values ± SD of 6 determinations for crispness and 25 for hardness. Different letters indicate significant differences (P<0.05) using ANOVA and Tukey tests. N, Newtons.
